# Current Trends in Bioethanol Production by *Saccharomyces cerevisiae*: Substrate, Inhibitor Reduction, Growth Variables, Coculture, and Immobilization

**DOI:** 10.1155/2014/532852

**Published:** 2014-12-08

**Authors:** Asmamaw Tesfaw, Fassil Assefa

**Affiliations:** Department of Microbial, Cellular and Molecular Biology, Addis Ababa University, P.O. Box 1176, Addis Ababa, Ethiopia

## Abstract

Bioethanol is one of the most commonly used biofuels in transportation sector to reduce greenhouse gases. *S. cerevisiae* is the most employed yeast for ethanol production at industrial level though ethanol is produced by an array of other yeasts, bacteria, and fungi. This paper reviews the current and nonmolecular trends in ethanol production using *S. cerevisiae*. Ethanol has been produced from wide range of substrates such as molasses, starch based substrate, sweet sorghum cane extract, lignocellulose, and other wastes. The inhibitors in lignocellulosic hydrolysates can be reduced by repeated sequential fermentation, treatment with reducing agents and activated charcoal, overliming, anion exchanger, evaporation, enzymatic treatment with peroxidase and laccase, *in situ* detoxification by fermenting microbes, and different extraction methods. Coculturing *S. cerevisiae* with other yeasts or microbes is targeted to optimize ethanol production, shorten fermentation time, and reduce process cost. Immobilization of yeast cells has been considered as potential alternative for enhancing ethanol productivity, because immobilizing yeasts reduce risk of contamination, make the separation of cell mass from the bulk liquid easy, retain stability of cell activities, minimize production costs, enable biocatalyst recycling, reduce fermentation time, and protect the cells from inhibitors. The effects of growth variables of the yeast and supplementation of external nitrogen sources on ethanol optimization are also reviewed.

## 1. Introduction

Industrialization and world population are continuously increasing and this demands high energy. As a result, the cost of crude oil, coal, and natural gas is increasing from time to time. Awareness of global climate change and the uncertainty of fossil fuel have thus led to the development of renewable energy. Biofuels are the renewable energy that gets attention these days. Bioethanol, biodiesel, and biogas are the dominant renewable energy among biofuels.

The most commonly used renewable fuel in the transportation sector is ethanol. Ethanol production worldwide has strongly increased since the oil crises in 1970. Its market grew from less than billion liters in 1975 to more than 39 billion liters in 2006, and it is expected to reach 100 billion liters in 2015 [1]. However, reduced production costs are essential to make liquid biofuels more competitive, especially when oil prices are below US$80 per barrel [2].

Ethanol can be produced from several substrates such as starch, lignocelluloses, and different wastes. However, almost all bioethanol is produced from grain or sugarcane at this time [3]. Lignocellulosic biomass is more preferred than starch or sugar-based crops for production of ethanol, since it does not compete with food and takes care of agricultural and plant residues in an environmentally sustainable process [4, 5]. For example, moderate increase in US corn ethanol production would result in modest changes in agricultural economies and net food insecurity; however, significant improvement in cellulosic ethanol production technology would substantially reduce the magnitude of such changes as increases in ethanol production could be fueled by previously unutilized agricultural wastes [6]. On the contrary, the argument that recent increases of biofuels production have a significant impact on feedstocks prices does not hold [7].

Industrial cellulosic ethanol production is still a challenge due to high processing cost. One reason for the high cost is the high steam energy consumption in the distillation of fermentation broth with low ethanol titer when lignocellulose materials are used as feedstock [36]. Nevertheless, economic ethanol can be produced from lignocellulosic substrates using* S. cerevisiae* [12]. For example, 0.21 g ethanol/1 g dry cellulosic feedstock is being produced with currently available technologies and this can be increased to at least 0.27 g ethanol/g biomass (83 g/ton) using simpler processes [37]. Another reason for high cost in ethanol production is higher feedstock prices [2, 38] whenever the substrate is noncellulose. Different pretreatment methods have evolved so far to increase the cellulose content in the fermentation system to upgrade ethanol titer [5, 10] and hence it reduces the cost.

The heart in ethanol production process is fermentation. Fermentation is carried out by a variety of microorganisms such as fungi, bacteria, and yeasts.* S. cerevisiae* is one of the widely studied and used yeasts at both industry and household levels.* S. cerevisiae* has generated ethanol as its main fermentation product.

*S. cerevisiae* is superior to bacteria, other yeasts, and filamentous fungi in various physiological characteristics regarding ethanol production in industrial context. It tolerates a wide range of pH [39] with acidic optimum [40], which makes its fermentation less susceptible to infection than bacteria. It also tolerates ethanol better than other ethanol producing microorganisms [8].* S. cerevisiae* is GRAS (generally regarded as safe) for human consumption which enhances its advantageous utilization more than other yeasts and microorganisms. This paper reviews the current trends of ethanol production using* S. cerevisiae* from different perspectives such as substrates, inhibitors reduction in biomass hydrolysates, growth variables, coculturing it with other microbes, and different immobilization techniques.

## 2. Substrate for* S. cerevisiae*

The substrates for ethanol production are recommended to be nonfood source and cheap. Ethanol has been produced from varieties of substrates (Table 1). First, ethanol production from different wastes such as molasses B [41], sugar beet pulp [42], waste from cassava starch production [20], food waste leachate [43], and waste newspapers [22] has been reported. Ethanol production from wastes has two major advantages. On the one hand, it reduces or eliminates cost of waste disposal. On the other hand, since wastes are cheap, it reduces cost of ethanol production. Second, nonfood extracts from sweet sorghum [28, 44, 45] and cashew apple [31] have been used as a substrate for* S. cerevisiae*. The extracts are best suited for ethanol production under very high gravity technology since adequate sugars are obtained through the extraction compared to lignocellulose hydrolysis. Third, chemicals like D-lactic acid [46] and inulin [47] have been utilized by* S. cerevisiae* and the yeast has produced promising amount of ethanol titer though producing lactic acid by itself is expensive for industrial level ethanol production. Fourth, the cheapest and lignocellulosic agricultural residues such as coffee pulp [16], coffee husk [25], corn stover [17, 19], sugarcane leaves [18], Jerusalem artichoke [47], rice hull [48], decorticated sorghum mash [49], cassava mash [50], cashew apple bagasse [26], mahula flowers [30], floriculture waste (*Dendranthema grandiflora*) [11], oil palm empty fruit bunches [12], oil seed rape straw [38], root biomass of* Coleus forskohlii* [51], mission grass (*Pennisetum polystachion*) [8], and rapeseed straw [52] were recently investigated to optimize lignocellulosic ethanol production.

The cellulose, hemicellulose, and lignin content of different plant residues vary and this results in varying ethanol concentration (Table 2, [53]). For example, the average percentage composition of cellulose, hemicellulose, and lignin of grasses from various provinces was determined as 31–38, 31–42, and 3–5, respectively [53]. The chemical composition (cellulose, hemicellulose, and lignin) of mission grass (*Pennisetum polystachion*) cultivated in different provinces (Tak Province and Nakhon Ratchasima Province, Thailand) was varied despite of the same species [8].

Bioethanol production from lignocellulose or starch requires chemical or/and biological pretreatment in order to be utilized by* S. cerevisiae* and as a result its production becomes costly and time demanding. A variety of researchers showed that chemical pretreatment increased the cellulose content (Table 2); however, the cellulose amount in biological pretreated lignocellulose was found to be lower than untreated one [5]. The cellulose utilization by organism as a carbon source to reproduce and grow may contribute significantly to reduction of cellulose in biological pretreatment. The total lignin was found to decrease after alkali pretreatment (Table 2) and this is due to easy solubility of lignin by alkali [12]. Different studies pointed out that chemical pretreatment had no consistence in hemicellulose content (Table 2).

During chemical pretreatment and treatment, five- and six-carbon sugars are produced in addition to cellobiose and partially degraded cellulose. Complete and efficient sugar utilization is one of the prerequisites for cost effective ethanol production from biomass.* S. cerevisiae* could not utilize the dominant five-carbon sugar, xylose, in biomass hydrolysate. Isomerization of xylose to xylulose has been used to solve such challenge. Another approach is to coculture five carbon utilizing and ethanol producing yeasts* like Pichia fermentans and Pichia stipitis* with* S. cerevisiae* [9, 10] so that both sugars can be efficiently utilized to optimize production process.

Kitchen wastes and ethanol stillage [50] served as substrate for* S. cerevisiae* after chemical and anaerobic microbial treatment, respectively. However, the organic acid particularly lactic acid present in kitchen wastes and anaerobically treated stillage hinders ethanol fermentation [50]. The problem was circumvented by using lactic acid as substrate for* S. cerevisiae* NAM34-4C and 2.7 g ethanol/L is produced from lactic acid at pH 3.0 and temperature 35°C [46]. The lactic acid assimilating* S. cerevisiae* NAM34-4C preferred minimal D-lactate rather than minimal L-lactate.

## 3. Inhibitor Reduction in Lignocellulosic Hydrolysates

Besides the five- and six-carbon sugars produced during hydrolysis of lignocellulosic biomass, several inhibitors of ethanol fermentation are also generated. For example, furans (furfural and hydroxymethylfurfural), carboxylic acids (acetic acids, levulinic acids, and formic acids), and phenolic compounds (syringaldehyde, 4-hydroxybenzaldehyde, catechol, vanillin, 4-hydroxybenzoic acid, dihydroconiferyl alcohol, coniferyl aldehyde, and syringic acid) are the most common inhibitors for ethanol production by* S. cerevisiae* from lignocellulosic hydrolysates [54, 55]. In addition to the three inhibitor categories, glycolaldehyde was reported as another inhibitory compound in lignocellulosic hydrolysates [56]. These inhibitors pose hindrances such as inhibition of cell growth and sugar consumption during* S. cerevisiae* cultivation for ethanol production [57].

Different approaches have been used to solve the inhibitory effects of these chemicals in the production process. For example, making the yeast adapt to the inhibitory chemicals with repeated sequential fermentation [58], treatment with reducing agents [59], addition of activated charcoal [57], overliming [8, 60], anion exchanger [55], evaporation [8], enzymatic treatment with peroxidase and laccase [61],* in situ* detoxification by fermenting microbes [52, 54], solvent extraction [61], and membrane extraction [62] have been investigated to reduce their inhibitory effects in ethanol fermentation. These approaches are categorized into biological, chemical, and physical methods.

The biological methods employ the use of enzymes or directly the microorganisms that detoxify the inhibitors in coculture or separate bases. The sequential coculturing of an extreme thermophilic bacterium* Thermoanaerobacter pentosaceus* and* S. cerevisiae* was investigated in alkaline-peroxide pretreated rapeseed straw to reduce inhibitory compounds and enhance ethanol production [52]. The result showed that* T*.* pentosaceus* was able to metabolize 5-hydroxymethyl furfural and furfural up to concentrations of 1 and 0.5 g/L, respectively. Likewise, phenolic compounds were detoxified using immobilized laccase from* Trametes versicolor* [55]. In addition, the coculture of* S. cerevisiae* Y5 and* P. stipitis* CBS6054 efficiently metabolized furfural and HMF and 0.46 g ethanol/g sugar was produced from nondetoxified dilute acid lignocellulosic hydrolysates [63].

The chemical treatment includes overliming, ion exchange, activated charcoal treatment, neutralization, and solvent extraction. The supplementation of activated charcoal to biomass hydrolysates reduced inhibitors [58] and it also shortened the fermentation time. Activated charcoals are best suited to remove inhibitors due to their high adsorption capacity [57]. More sugar uptake was also observed besides enhanced ethanol production in media treated with activated charcoal compared to nontreated ones.

Treatment with reducing agent and lime are other chemical methods that get attention despite the uneconomic reducing agent at industry level due to its cost. The fermentability of inhibitory lignocelluloses was improved using reducing agents like dithionite and sulfite [59]. The addition of dithionite to enzymatically hydrolyzed spruce wood and sugarcane bagasse increased ethanol production from 0.2 to 2.5 g/L/h and the bagasse hydrolysate from 0.9 to 3.9 g/L/h, respectively, whereas the addition of sulfite increased ethanol production from 0.2 to 1.2 and 0.9 to 2.9 g/L/h, respectively, under separate saccharification and fermentation condition. Although the way how overliming detoxifies lignocellulosic inhibitors is still not clearly understood, the detoxification might be due to precipitation of toxic compounds in the hydrolysates or/and chemical conversion at high pH [60]. Precipitation technique reduces acetic acid and levulinic acid in the hydrolysates by neutralization chemistry principle.

Evaporation and membrane separation are the most commonly used physical methods to reduce inhibitory chemicals in biomass hydrolysates. Volatile inhibiting compounds such as furfural, acetic acids, formic acids, and other lignin degradation products were reduced by evaporation in lignocellulosic hydrolysates [8, 54]. Though evaporation is less costly and eases operation [8], it requires a lot of energy and this might make it uneconomical at industry level. Evaporation is also used to regulate sugar concentration in hydrolysates besides inhibitory reduction [54].

Organic solvent extraction of the inhibitors using n-butanol, trialkylamine, and ethyl acetate decreased the inhibitors greatly [57, 61]. However, conventional solvent extraction methods have some limitations as follows: mixing of one phase in the other, emulsifying challenges, loading and flooding problems, and scaling-up difficulty. These limitations are overcome by membrane extraction [62]. In addition, by eliminating the need to disperse one phase in the other, subsequent coalescence of the dispersed phase is also eliminated. Furthermore, membrane extraction is best suited to extract acetic acid that cannot be removed easily by solvent extraction methods [62]. Nevertheless, 73.3% of acetic acid was removed from corn stover prehydrolyzate using trialkylamine extraction method [61]. During membrane extraction, modification of polypropylene membrane bases via deposition of polyelectrolytes enabled removing a variety of kinds of inhibitors in pretreated biomass [62]. These properties make membrane extraction superior over solvent extraction. However, solvent extraction may be better than membrane extraction in the context that there is a possibility of recycling the solvent to make the detoxification more economical.

Synergistic effects of detoxification methods have been investigated in several studies. For instance, overliming and then sodium sulfite methods [8], overliming and then adsorption onto ion-exchange resins [64], and overliming and then activated charcoal methods [23] further reduced the inhibitor compounds compared to one method alone.

## 4. Coculturing* S. cerevisiae* with Other Microbes for Enhanced Ethanol Production

Coculture is a mimic of natural environment [65] and it is a potential bioprocess if there are no cross interactions among themselves for substrate utilization and by toxin production [65, 66]. At high glucose/xylose concentration (50/20 g/L), glucose is primarily utilized whereas at low mixture concentration (25/10) simultaneous consumption of sugars was observed [4]. The* S. cerevisiae* cell utilized its own carbohydrate reserve instead of xylose when glucose was consumed in lignocellulosic hydrolysates containing xylose [60]. Researchers have been investigating various means to solve this problem. On the one hand, isomerization of the xylose using isomerase reduced the xylose in the hydrolysates and upgraded ethanol production [60]. On the other hand, coculturing the* S. cerevisiae* that prefer six-carbon sugars with yeasts that produce efficient ethanol from five-carbon sugars is also another alternative to optimize ethanol in hydrolysates containing xylose [4, 9, 10].

Coculture of* S. cerevisiae* ITV-01 and* Pichia stipitis* NRRL Y-7124 was investigated by Gutiérrez-Rivera et al. [4]; they found that ethanol productivity increased fivefold compared to monocultures. This improvement in ethanol productivity might be due to enhanced substrate utilization since* S. cerevisiae* uses the six-carbon (glucose) source and* P. stipitis* uses the five-carbon (xylose) source to produce ethanol. However, the problem in this coculture was that* P. stipitis* NRRL Y-7124 tolerated lower ethanol inhibition than* S. cerevisiae ITV-01* and hence the ethanol concentration produced by* S. cerevisiae *ITV-01 prevented further ethanol production in* P. stipitis* NRRL Y-7124 [4]. Similarly, the coculturing of* S. cerevisiae* MTCC 174 and* Scheffersomyces stipitis* NCIM No. 3497 (formerly* P. stipitis*) was studied using microwave alkali pretreated rice husk medium [9]; it was reported that their coculture produces maximum ethanol concentration (20.8 g/L) compared to* S. cerevisiae* MTCC 174 (14.0 g/L) and* S. stipitis* NCIM No. 3497 (12.2 g/L) alone. Likely, more ethanol was produced in* S. cerevisiae* ATCC 26602 and* S. stipitis* DSM 3651 coculture (7.36 g/L) compared to* S. cerevisiae* monoculture (6.68 g/L) using H_2_O_2_ pretreated and enzyme hydrolyzed wheat straw [10]. Generally, increased ethanol production might be contributed to the competition of* S. stipitis* for xylose though the mechanism was not shown in their investigation [10].

In addition to* S. cerevisiae* and* P. stipitis* coculture, concentrated ethanol (75.37 g/L) and the lowest levels of residual glucose (1.14 g/L) were found in the mixture of* Pichia caribbica* UFLA CAF733 and* S. cerevisiae* UFLA CA11 in the sugar cane spirit (cachaca) fermentation process [67]. In another study,* Candida shehatae* HM 52.2 was cocultured with* S. cerevisiae* ICV D254 in synthetic medium and rice hull hydrolysate and the result demonstrated that the coculture was effective in simultaneously converting glucose and xylose, maximizing substrate utilization rates, increasing ethanol yields and production rates [48]. This coculture was found to be inhibited by the rate of oxygenation and furanic inhibitors in the medium.

*S. cerevisiae* has been cocultured with polysaccharide solubilizing microorganisms to get simple sugars for ethanol fermentation. The cocultural condition of cellulase producing* Acremonium cellulolyticus* with ethanol producing* S. cerevisiae* was studied using Solka-Floc as cellulase-inducing substrate under one-pot process in single reactor; the ethanol was maximized to 46.3 g/L [65] and it can be concluded that it is promising to produce ethanol without pretreatment and extraneous cellulase. In the same manner, more ethanol from starch was produced when amylolytic yeasts* Saccharomyces diastaticus* and* Endomycopsis capsularis* mixed with* S. cerevisiae *21 compared to their respective monocultures [66]. Therefore, coculturing reduces the cost required for chemical pretreatment and extraneous enzymes.

Generally, coculturing* S. cerevisiae* with other microbes reduces inhibitory compounds in lignocellulosic hydrolysates [52, 54, 63], increases ethanol yield and production rate [9, 63], shortens fermentation time, and reduces process cost [48, 66]. Therefore, coculturing could be an alternative strategy for ethanol production besides the classical way of biofuel optimization.

## 5. Growth Variables Affecting Ethanol Fermentation

Temperature, pH, oxygen, initial sugar concentrations, organic acids, dissolved solids, and immobilization of the yeast are greatly essential parameters that influence the specific rate of yeast growth and ethanol production. Medium conditions direct the viability of yeasts, specific rate of fermentation, and nutrient uptake [39].

### 5.1. Temperature

Temperature greatly affects the enzymatic activity and membrane turgidity of yeast cells and yeasts which are active and tolerant at high temperature are ideal for industrial bioethanol production.* S. cerevisiae* ITV-01 yeast, isolated from sugar cane molasses, was found to produce more ethanol (58.4 g/L) optimally at 30°C with pH 3.5 [40]. In the other study, 30–40°C were optimal for* S. cerevisiae* BY4742; higher temperature shortened the exponential phase of the yeast cell [39]. Ethanol production reduced considerably at 50°C and this might be due to change in transport system which might increase accumulation of toxin including ethanol in the cell [39]. In addition, enzymes and ribosome denaturation and membrane fluidity problems might be brought by higher temperature. Though 30–35°C were best for yeast strain fermentation,* S. cerevisiae* JZ1C inulinases function efficiently at the temperature range between 40 and 50°C [47]. Therefore, the yeast should be active and tolerant at higher temperature to produce ethanol using inulin as a carbon source. In the other study, ethanol production decreased when the temperature was raised to 30°C using alkali pretreated palm fruit bench fiber under fed-batch SSF condition [12]; uneconomical ethanol was produced at 37°C and higher.

### 5.2. pH

Optimum pH for* S. cerevisiae* BY4742 was in the range of 4.0–5.0 [39]; when the pH was lower than 4.0, the incubation period was prolonged though the ethanol concentration was not reduced significantly and when the pH was above 5.0, the concentration of ethanol diminished substantially. Formation of acetic acid was enhanced when the pH was below 4.0 and pH above 5.0 favored butyric acid productions [39]. Unlikely, pH 3.5 was optimal for ethanol production by* S. cerevisiae* ITV-01 at 30°C with initial glucose concentration of 150 g/L [40]. A wide range of optimum pH (4.0–8.0) was reported for* S. cerevisiae* JZ1C isolated from rhizosphere of Jerusalem artichoke using inulin and Jerusalem artichoke tuber as substrate at 35°C [47].

Currently, stillage (a waste after ethanol production) is commonly reused for yeast substrate to make the ethanol production more efficient; however, stillage contains more organic acids than expected. The organic acids present in the stillage elongated the ethanol fermentation time [50]; ethanol fermentation from cassava mash using* S. cerevisiae* was more inhibited by propionic acid as medium pH decreased, undissociated acid being the effective inhibitory form, whereas glycerol production decreased as propionic acid increased irrespective of solids in cassava mash and pH condition. The plasma membrane allows the easy entrance of undissociated acids, dissociating intracellularly and thus cytoplasm could be acidified. At the same time, the proton must be transported by membrane ATPase to maintain intracellular pH and thus it results in increased ATP consumption and decreased biomass yield [50].

The above discussion shows that different acids produced by the yeast or added exogenously created optimum pH or unfavorable pH range for the* S. cerevisiae.* On the other hand, different investigations proved that yeast uses organic acids as a substrate.* S. cerevisiae* NAM34-4C grew rapidly and produced ethanol (2.7 g/L) in YPDL (10, yeast extract; 20, peptone; and 20, D-lactic acid g/L) medium at pH 3.5 and temperature 35°C [46]. Similarly, the volatile acidity from acidic white wine was efficiently reduced by* S. cerevisiae* S26 when the acetic acid and ethanol concentration were kept below 1.0 g/L and 11% (v/v), respectively [68].

### 5.3. Initial Sugar Concentration

The effect of initial reducing sugar concentration from sweet sorghum stalk juice on* S. cerevisiae* CICC 1308 immobilized with sodium alginate was studied [44]. Accordingly, when initial sugar concentration was increased, the average specific growth rate and average biomass yield were significantly inhibited whereas average specific substrate uptake, average specific ethanol productivity, and average ethanol yield were increased (sugar concentration in the range of 85–156 g/L at 30°C was evaluated). Similarly, as reducing sugar concentration obtained from food waste leachate was increased from 45 to 75 g/L to grow* S. cerevisiae* KCTC-7904, the ethanol production was raised in 2.3-fold [43]. Ethanol yields were reported to increase with increasing glucose concentration (from 15 to 60 g/L) using* S. cerevisiae* immobilized with Lentikat discs in continuous flow packed bed columns [38]. Unlike the above studies, low amount of ethanol (0.22 L ethanol kg^−1^) was produced at higher gravity sorghum mashes (20°Plato) than lower counterpart (13°Plato) that produced 0.22 L ethanol kg^−1^ [49].

### 5.4. Supplementation of External Nitrogen Sources and Growth Factors

The supplementation of exogenous nitrogen sources such as yeast extract, malt extract, peptone, and (NH_4_)_2_SO_4_ to the natural growing media enhanced ethanol production in* S. cerevisiae* [49, 51]. Supplements also enhance sugar utilization [19, 40, 49, 51, 69] which might be one reason for better ethanol yield with supplemented substrates. One reason for enhanced ethanol production with yeast extract supplementation was the presence of important cofactors like biotin and riboflavin [40].* S. cerevisiae* Y5 nitrogen source (corn steep liquor (CSL), yeast extract, and peptone) preference was evaluated in enzymatic hydrolysate of nondetoxified steam-exploded corn stover for ethanol production and it was found that higher ethanol was produced in CSL (44.55 g/L ethanol, corresponding to 94.5% of the theoretical value) compared to yeast extract and peptone (40.89 g/L ethanol, corresponding to 86.7% of the theoretical value); glucose consumption with yeast extract and peptone (glucose depletion in 36 hrs) as the nitrogen source was significantly lower than that with CSL (12 hrs) [19]. The better result in CSL is probably by the presence of nutrients in CSL but absent in the other formulations and CSL is generally rich in nitrogen, water soluble vitamins, amino acids, minerals, and other stimulants [19]. Likely, the addition of acid hydrolyzed bloom algae powder as nitrogen supplementation under high gravity technology improved ethanol production (104.3 g/L) and shortened fermentation time [15].

On the contrary, supplementation of (NH_4_)_2_SO_4_, yeast extract, and distillers' dried grains with solubles (DDGS) to the SSF of pretreated corn stover with dilute H_2_SO_4_ did not bring any change on ethanol yield using thermotolerant strain* S. cerevisiae* DQ1 [17]; however, ethanol production was found higher when the cellulase dosage increased (until 15 FPU/gDM) at a temperature below 37°C. On the other hand, ethanol amount reduced when the cellulase dose increased at 40°C or above; the reason for ethanol production is most probably the decomposition of cell wall by cellulase at higher temperature [17]. Unlike [17], the addition of DDGS to corn stover hydrolysate enhanced ethanol production to the extent of the expensive yeast extract using* S. cerevisiae* DQ1 in SSF condition [21].

In another contradiction, (NH_4_)_2_SO_4_ supplementation to the hydrolysate of cassava pulp (a waste from cassava starch production) did not enhance ethanol production by the yeast [20]. Another contradiction from [41] reported that the addition of yeast extract, ammonium sulfate, urea, and their combination to molasses B (sugar rich molasses obtained during the second step of crystallization) did not improve ethanol productivity significantly.

Like [19, 49], higher ethanol yield could be obtained from the addition of vitamins, amino acids, sterols, or yeast extract [69]. However, these supplements are too expensive to use at industrial level and hence cheap additives such as sunflower, groundnut, and safflower oilseed meal cakes, wheat mash, or soy flour could be used. Despite higher ethanol in all supplemented media compared to unsupplemented media, safflower oilseed meal cake provided higher ethanol than the rest [69]. It is well known that safflower oilseed contains polyunsaturated fats and unsaturated fatty acids played a great role in ethanol tolerances [70]; this might lead to enhanced ethanol production. Similarly, supplementation of oilseed meal (4%) from safflower enhanced the ethanol production by 50% and the sugar tolerance was improved from 8 to 16%; the addition of 2% (g/V) rice husk also raised ethanol amount by 48% [69]. This might lead to concluding that some sort of nutrients present in safflower might enhance the yeast metabolism towards better ethanol yield.

Slight decrease, pronounced decrease, and almost complete inhibition of fermentation rate and ethanol production were found by the addition of 0.18, 0.72, and 2.16% (w/v) of calcium as calcium chloride to molasses [71] though its effect was minimized by molasses pretreatment with sulfuric acid and the calcium precipitate after cooling the treated molasses; the decrease in ethanol yield might be, in part, due to invertase inhibition by calcium.

### 5.5. Inoculum Size

Lower inoculum size reduces cost of production in ethanol fermentation. For instance, 5% (v/v) and 12 hrs old inoculum sizes yielded almost the same result with 10% using* S. cerevisiae* Y5 in enzymatic hydrolysate of nondetoxified steam-exploded corn stover supplemented with CSL [19]. Ethanol productivity by baker yeast decreased as yeast concentration increased from 3 to 4 and 5 g/L in coffee husk based substrate [25]. However, 10% (v/v)* S. cerevisiae* TISTR 5596 was used to produce high ethanol using waste from cassava starch production without nitrogen source supplementation [20].

Correspondingly, pronounced increment in both substrate utilization and ethanol production rates was found at high initial concentration of a recombinant, flocculent, and five-carbon sugar utilizing* S. cerevisiae* MA-R4 in a medium that contains both xylose and glucose; however, it had no positive effect on ethanol yield mainly due to accumulated by-products including xylitol [72]. Differently, the effect of inoculum size on ethanol yield was studied by [73] using response surface methodology and it was found that raised ethanol yields were obtained with high inoculum size. The ethanol production was raised from 1.29 to 2.35 g/L/h when the yeast load increased from 0.5 to 5 g/L by shortening the lag phase in fed-batch separate saccharification and fermentation (SSF) process though the study did not report on the effect of yeast loading greater than 5 g/L yeast [12].

## 6. Immobilization Improves Ethanol Productivity

The most commonly used immobilizing agents are sodium or calcium alginate and agar-agar cubes [10]. Alternatively, new immobilizing agents that are cheap and easy to use have been investigated in several studies (Table 3). These include sugarcane bagasse [27], alginate-chitosan beads [32, 74], corncob pieces [28], sweet sorghum pith [29], alginate-maize stem ground tissue matrix [33], cashew apple bagasse [31], lyophilized cellulose gel [34], dried spongy fruit of luffa (*Luffa cylindrica* L.) [30], carboxymethylcellulose (CMC) grafted with N-vinyl-2-pyrrolidone [75], sodium alginate grafted with N-vinyl-2-pyrrolidone [35], Lentikat discs [38], and rice flour and white glutinous rice flour [76].

Immobilization of yeast cells has been considered as potential alternative for enhancing ethanol productivity, because immobilizing yeasts reduce risk of contamination [33, 76], make the separation of cell mass from the bulk liquid easy [76], retain stability of cell activities [77], minimize production costs [24, 27, 31], enable biocatalyst recycling [76], reduce fermentation time [10, 24], and protect the cells from inhibitors [77].

In addition, immobilizing* S. cerevisiae* S26 potentially reduced the volatile acidity of acidic wines without affecting the aroma of the wine since high acetic acid brought undesirable acidic taste and unpleasant vinegar aroma to wine [74]. Immobilized yeast cells were found to be superior to the free yeast cells since immobilized cells are more tolerant to ethanol and lower substrate inhibition [24]. Different researchers concluded that immobilized* S. cerevisiae* produced more ethanol compared to free cells [10, 24, 30, 33] though the immobilizing agents used were different (Table 3).

Initial yeast cell concentration was found to be determinant in ethanol production with immobilization; higher sugar consumption and ethanol production rate were observed at higher initial yeast cell concentration [28, 45]. On the contrary, the maximum final ethanol concentration, ethanol yield, and volumetric productivity were obtained at 2% (w/v) initial concentration with 176 g/L initial glucose concentration compared to 10 and 20% [24]. This entails that there is no need to add higher initial yeast concentration since concentrated yeast cells did not lead to higher ethanol concentration. The reason might be the depletion of sugars at higher initial sugar concentration.

Recycling microorganisms saves time, energy, and money whenever they are applied properly especially at industrial level. As a result, a variety of researches have been investigated on yeast recycling. The immobilized yeast cells were found to be reusable for 15 cycles with bacterial cellulose-alginate sponge [77], 10 cycles under very high gravity fermentation [29, 31], 10 cycles with decrement of ethanol concentration after 7 cycles [27], using sugar cane bagasse as a supporting material, 4 cycles in carboxymethylcellulose [75], 3 cycles in lyophilized cellulose gel [34], and 3 cycles in luffa spongy discs [30].

Generally, recently investigated supporting materials are better than the classical immobilizing agents to produce ethanol since they are cheap and easy to use. For instance, lignocellulose based immobilizing materials gave way enhanced ethanol compared to the commonly used supporting material like sodium or calcium alginate [27]. In addition,* S. cerevisiae* immobilized by sodium alginate grafted with N-vinyl-2-pyrrolidone produced more ethanol than sodium alginate alone [35].

## 7. Conclusion and Future Directions

Ethanol has been produced from molasses and starch for long period of time; however, ethanol production from starch leads competition for food regarding land and price. Therefore, lignocellulosic agricultural residues are potentially used for ethanol production to solve such challenges. Nevertheless, its industrial production is not successful due to low ethanol titer and different inhibitors in lignocellulosic hydrolysates. The low ethanol titer is circumvented using a variety of optimization techniques. Overliming, solvent and membrane extractions, adsorption with activated charcoal, and treatment with reducing agents potentially reduce the inhibitors to get higher ethanol liter. Coculturing* S. cerevisiae* with other microbes enhances its production from different perspectives. Immobilizing the yeast with cheap supporting materials is another strategy to optimize the production process in less cost manner. Therefore, lignocellulose pretreatment and the yeast fermentation technology are still an area of research interest for the second generation fuel production. In current day molecular era, transformation and overexpression of a gene related to specific traits (e.g., cellulase) in* S. cerevisiae* might be very important to solve challenges like inability to utilize polysaccharide and ribose. Therefore, a comprehensive economic and process analysis is required to develop an industrially suitable production strategy that will solve our energy crisis by producing more ethanol in a stable way [78].

## Figures and Tables

**Table 1:** Ethanol production by *S. cerevisiae* from different substrates at varying treatment and optimization conditions.

*S. cerevisiae* strain	Substrate	Pretreatment	Treatment method	Enzymatic hydrolysis	Ethanol produced (g/L)	References
TISTR 5596	Mission grass	NaOH	H_2_SO_4_		16^E^	[8]

MTCC 174	Rice husk	NaOH		Crude unprocessed enzyme	14	[9]

ATCC 26602	Wheat straw	H_2_O_2_		cellulase	10	[10]

SOL/M5	Leaf and stem of *Dendranthema grandiflora *			Crude extract from *Pleurotus ostreatus *	10.64	[11]

L2524a	Empty palm fruit bunch fibers	Alkali (NaOH)		Cellulase	64.2^B^	[12]

TJ14	Microcrystalline cellulose			Commercial cellulase	45^B^	[13]

Y5	Corn stover	Steam explosion		Cellulase and *β*-glucosidase	50^B^	[14]

ATCC 6508	Sweet potato chips			*α*-Amylase and glucoamylase	104.3^D^	[15]

Baker yeast	Coffee pulp		Hydrolysis by H_2_SO_4_		7.4	[16]

DQ1	Corn stover	H_2_SO_4_ supplemented with hexadecyl trimethyl ammonium bromide		Cellulase	48^B^	[17]

TISTR 5596	Sugarcane leaves	^ A^H_2_SO_4 _ or Ca(HO)_2_		cellulase	4.71	[18]

Y5	Corn stover	Steam explosion		cellulase	40	[19]

TISTR 5596	starch cassava pulp			*α*-amylase and glucoamylase	9.9	[20]

TISTR 5596	lignocellulosic fiber in cassava pulp	^ A^H_2_SO_4_ or Ca(OH)_2_		Cellulase	11.9	[20]

DQ1	Corn stover ^C^	steam explosion		Cellulase	55^B^	[21]

ATCC 96581	Waste newspaper	sodium dodecyl sulphate		Cellulase and *β*-glucosidase	14.29	[22]

RCK-1	newspaper cellulosics			exoglucanase, *β*-glucosidase and xylanases with Tween 80 and CoCl_2_	5.64 (batch) and 14.77 (fed batch)	[23]

var. *ellipsoideus *	Corn meal			Heat stable *α*-amylase and glucoamylase	79.6^F^	[24]

Baker yeast	Sticky coffee husks				13.6	[25]

A: at 121°C and 2 atm; B: simultaneous saccharification and fermentation; C: supplemented with dry distiller's grain and solubles; D: acid hydrolyzed bloom algae powder was added under very high gravity condition (210 g/L glucose); E: Overlimed at pH 10; and F: the yeast was immobilized and the sugar concentration was 87.6 g/L.

**Table 2:** Composition of some lignocellulosic biomass (in percentage).

*S. cerevisiae* strain	Substrate	Pretreatment	Composition (A) of substrates before and after pretreatment						Ethanol produced (g/L)	References
			Cellulose		Hemicellulose		Lignin	
			Before	After	Before	After	Before	After
CBS 8066	Oil palm empty fruit bunches	H_3_PO_4_ and fungi	39.13	53.81	23.04	9.07	34.37	37.22	23 (B)	[5]

TISTR 5596	Thai Mission grass	NaOH	47.2		27.3		18.2		16 (C)	[8]

ATCC 26602	Wheat straw	H_2_O_2_	42.8	63.5	23.8	23.6	15.1	9.1	10	[10]

MCAB-H	Cashew apple bagasse	H_2_SO_4_	20.9	27.2	16.3	5.3	33.6	50.3	9.59	[26]

L3262a	Empty palm fruit bunch fibers	NaOH	39.8	58.0	17.3	21.1	28.8	8.8	62.5 (D)	[12]

MTCC 174	Sugar cane bagasse	NaOH	43	55.2	24	31.6	20	8.3	15.4	[27]

(A) The extractives and ashes are included in compositional analysis; (B) there was additional pretreatment with white-rot fungus *Pleurotus floridanus*; (C) the hydrolysates were overlimed at pH 10; and (D) the fermentation was carried out under simultaneous saccharification and fermentation (SSF) with cellulase and yeast.

**Table 3:** Immobilizing agents to enhance ethanol production using different *S. cerevisiae* strains and substrates.

*S. cerevisiae* strain	Substrate	Initial sugar (g/L)	Residual sugar (g/L)	Immobilizing materials	Ethanol produced (g/L)	Ethanol yield (g/g)	References
MTCC 174	Sugar cane bagasse	50	15	Sugar cane bagasse	15.4	0.44	[27]

MTCC 174	Sugar cane bagasse	50	22	Agar-agar cubes	9.4	0.33	[27]

TISTR 5048	Sweet sorghum juice	240	26.69	Corncobs	102.39	0.48	[28]

NP 01	Sweet sorghum juice	240	54.8	Corncobs	90.75	0.49	[28]

Mutant baker yeast 3013	Glucose + sucrose	280	7.21	Sweet sorghum pith	130.12	0.477	[29]

CTCRI	Mahula flowers	89.75	7.99	Luffa sponge discs	37.2	0.455	[30]

Baker yeast	Cashew apple juice	70.01	3.92	Cashew apple bagasse	36.91	0.49	[31]

CBS 8066	Glucose	30	0.3	Alginate-chitosan beads	13.37	0.45	[32]

DTN	Sugar beet molasses	130		Alginate-maize stem ground tissue	60.36	0.493	[33]

Baker yeast	Glucose	100	16	Lyophilized cellulose gel	36.12	0.43	[34]

Pakmaya Yeast Company	Glucose			Sodium alginate grafted with N-vinyl-2-pyrrolidone	69.68	0.697	[35]

*Saccharomyces cerevisiae* var. *ellipsoideus *	Corn meal hydrolysates	176	8.02	Calcium alginate	89.68	0.52	[24]

## References

[1] Licht F. (2006). *World Ethanol Markets: The Outlook to 2015*.

[2] Timilsina G. R., Shrestha A. (2011). How much hope should we have for biofuels?. *Energy*.

[3] Mussatto S. I., Dragone G., Guimarães P. M. R. (2010). Technological trends, global market, and challenges of bio-ethanol production. *Biotechnology Advances*.

[4] Gutiérrez-Rivera B., Waliszewski-Kubiak K., Carvajal-Zarrabal O., Aguilar-Uscanga M. G. (2012). Conversion efficiency of glucose/xylose mixtures for ethanol production using *Saccharomyces cerevisiae ITV01* and *Pichia stipitis* NRRL Y-7124. *Journal of Chemical Technology and Biotechnology*.

[5] Ishola M. M., Taherzadeh M. J. (2014). Effect of fungal and phosphoric acid pretreatment on ethanol production from oil palm empty fruit bunches (OPEFB). *Bioresource Technology*.

[6] Bryant H. L., Lu J., Richardson J. W., Outlaw J. L., Schmitz A., Moss C. B. (2011). Outlaw, long-term effects of increasing ethanol production on agricultural markets and trade, land use, and food security. *The Economics of Alternative Energy Sources and Globalization*.

[7] Ajanovic A. (2011). Biofuels versus food production: does biofuels production increase food prices?. *Energy*.

[8] Prasertwasu S., Khumsupan D., Komolwanich T., Chaisuwan T., Luengnaruemitchai A., Wongkasemjit S. (2014). Efficient process for ethanol production from Thai Mission grass (*Pennisetum polystachion*). *Bioresource Technology*.

[9] Singh A., Bajar S., Bishnoi N. R. (2014). Enzymatic hydrolysis of microwave alkali pretreated rice husk for ethanol production by *Saccharomyces cerevisiae*, *Scheffersomyces stipitis* and their co-culture. *Fuel*.

[10] Karagoz P., Ozkan M. (2014). Ethanol production from wheat straw by *Saccharomyces cerevisiae* and *Scheffersomyces stipitis* co-culture in batch and continuous system. *Bioresource Technology*.

[11] Quevedo-Hidalgo B., Monsalve-Marín F., Narváez-Rincón P. C., Pedroza-Rodríguez A. M., Velásquez-Lozano M. E. (2013). Ethanol production by *Saccharomyces cerevisiae* using lignocellulosic hydrolysate from *Chrysanthemum* waste degradation. *World Journal of Microbiology and Biotechnology*.

[12] Park J. M., Oh B. R., Seo J. W. (2013). Efficient production of ethanol from empty palm fruit bunch fibers by fed-batch simultaneous saccharification and fermentation using *Saccharomyces cerevisiae*. *Applied Biochemistry and Biotechnology*.

[13] Shahsavarani H., Hasegawa D., Yokota D. (2013). Enhanced bio-ethanol production from cellulosic materials by semi-simultaneous saccharification and fermentation using high temperature resistant *Saccharomyces cerevisiae* TJ14. *Journal of Bioscience and Bioengineering*.

[14] Tian S., Li Y., Wang Z., Yang X. (2013). Evaluation of simultaneous saccharification and ethanol fermentation of undetoxified steam-exploded corn stover by *Saccharomyces cerevisiae* Y5. *Bioenergy Research*.

[15] Shen Y., Guo J.-S., Chen Y.-P. (2012). Application of low-cost algal nitrogen source feeding in fuel ethanol production using high gravity sweet potato medium. *Journal of Biotechnology*.

[16] Kefale A., Redi M., Asfaw A. (2012). Potential of bioethanol production and optimization test from agricultural waste: the case of wet coffee processing waste (pulp). *International Journal of Renewable Energy Research*.

[17] Chu D., Zhang J., Bao J. (2012). Simultaneous saccharification and ethanol fermentation of corn stover at high temperature and high solids loading by a thermotolerant strain *Saccharomyces cerevisiae* DQ1. *Bioenergy Research*.

[18] Jutakanoke R., Leepipatpiboon N., Tolieng V., Kitpreechavanich V., Srinorakutara T., Akaracharanya A. (2012). Sugarcane leaves: pretreatment and ethanol fermentation by *Saccharomyces cerevisiae*. *Biomass and Bioenergy*.

[19] Li Y., Gao K., Tian S., Zhang S., Yang X. (2011). Evaluation of *Saccharomyces cerevisiae* Y5 for ethanol production from enzymatic hydrolysate of non-detoxified steam-exploded corn stover. *Bioresource Technology*.

[20] Akaracharanya A., Kesornsit J., Leepipatpiboon N., Srinorakutara T., Kitpreechavanich V., Tolieng V. (2011). Evaluation of the waste from cassava starch production as a substrate for ethanol fermentation by *Saccharomyces cerevisiae*. *Annals of Microbiology*.

[21] Bi D., Chu D., Zhu P. (2011). Utilization of dry distiller's grain and solubles as nutrient supplement in the simultaneous saccharification and ethanol fermentation at high solids loading of corn stover. *Biotechnology Letters*.

[22] Xin F., Geng A., Chen M. L., Gum M. J. M. (2010). Enzymatic hydrolysis of sodium dodecyl sulphate (SDS)—pretreated newspaper for cellulosic ethanol production by *Saccharomyces cerevisiae* and Pichia stipitis. *Applied Biochemistry and Biotechnology*.

[23] Kuhad R. C., Mehta G., Gupta R., Sharma K. K. (2010). Fed batch enzymatic saccharification of newspaper cellulosics improves the sugar content in the hydrolysates and eventually the ethanol fermentation by *Saccharomyces cerevisiae*. *Biomass and Bioenergy*.

[24] Nikolić S., Mojović L., Pejin D., Rakin M., Vukašinović M. (2010). Production of bioethanol from corn meal hydrolyzates by free and immobilized cells of *Saccharomyces cerevisiae* var. ellipsoideus. *Biomass and Bioenergy*.

[25] Gouvea B. M., Torres C., Franca A. S., Oliveira L. S., Oliveira E. S. (2009). Feasibility of ethanol production from coffee husks. *Biotechnology Letters*.

[26] Rocha M. V. P., Rodrigues T. H. S., de Albuquerque T. L., Gonçalves L. R. B., de Macedo G. R. (2014). Evaluation of dilute acid pretreatment on cashew apple bagasse for ethanol and xylitol production. *Chemical Engineering Journal*.

[27] Singh A., Sharma P., Saran A. K., Singh N., Bishnoi N. R. (2013). Comparative study on ethanol production from pretreated sugarcane bagasse using immobilized *Saccharomyces cerevisiae* on various matrices. *Renewable Energy*.

[28] Laopaiboon L., Laopaiboon P. (2012). Ethanol production from sweet sorghum juice in repeated-batch fermentation by *Saccharomyces cerevisiae* immobilized on corncob. *World Journal of Microbiology and Biotechnology*.

[29] Ji H., Yu J., Zhang X., Tan T. (2012). Characteristics of an immobilized yeast cell system using very high gravity for the fermentation of ethanol. *Applied Biochemistry and Biotechnology*.

[30] Behera S., Mohanty R. C., Ray R. C. (2011). Ethanol production from mahula (*Madhuca latifolia* L.) flowers with immobilized cells of *Saccharomyces cerevisiae* in *Luffa cylindrica* L. sponge discs. *Applied Energy*.

[31] Pacheco A. M., Gondim D. R., Gonçalves L. R. B. (2010). Ethanol production by fermentation using immobilized cells of *Saccharomyces cerevisiae* in cashew apple bagasse. *Applied Biochemistry and Biotechnology*.

[32] Ylitervo P., Franzén C. J., Taherzadeh M. J. (2011). Ethanol production at elevated temperatures using encapsulation of yeast. *Journal of Biotechnology*.

[33] Razmovski R., Vučurović V. (2011). Ethanol production from sugar beet molasses by *S. cerevisiae* entrapped in an alginate-maize stem ground tissue matrix. *Enzyme and Microbial Technology*.

[34] Winkelhausen E., Velickova E., Amartey S. A., Kuzmanova S. (2010). Ethanol production using immobilized *Saccharomyces cerevisiae* in lyophilized cellulose gel. *Applied Biochemistry and Biotechnology*.

[35] Inal M., Yiğitoğlu M. (2011). Production of bioethanol by immobilized *Saccharomyces cerevisiae* onto modified sodium alginate gel. *Journal of Chemical Technology and Biotechnology*.

[36] Nikolić S., Mojović L., Djukić-Vuković A., Dincer I., Colpan C. O., Kadioglu F. (2013). Possibilities of improving the bioethanol production from cornmeal by yeast *Saccharomyces cerevisiae* var. *ellipsoideus*. *Causes, Impacts and Solutions to Global Warming*.

[37] Geddes C. C., Nieves I. U., Ingram L. O. (2011). Advances in ethanol production. *Current Opinion in Biotechnology*.

[38] Mathew A. K., Crook M., Chaney K., Humphries A. C. (2014). Continuous bioethanol production from oilseed rape straw hydrosylate using immobilised Saccharomyces cerevisiae cells. *Bioresource Technology*.

[39] Lin Y., Zhang W., Li C., Sakakibara K., Tanaka S., Kong H. (2012). Factors affecting ethanol fermentation using *Saccharomyces cerevisiae* BY4742. *Biomass and Bioenergy*.

[40] Ortiz-Muñiz B., Carvajal-Zarrabal O., Torrestiana-Sanchez B., Aguilar-Uscanga M. G. (2010). Kinetic study on ethanol production using *Saccharomyces cerevisiae* ITV-01 yeast isolated from sugar canemolasses. *Journal of Chemical Technology and Biotechnology*.

[41] Fernández-López C. L., Torrestiana-Sánchez B., Salgado-Cervantes M. A., Mendoza García P. G., Aguilar-Uscanga M. G. (2012). Use of sugarcane molasses “B” as an alternative for ethanol production with wild-type yeast *Saccharomyces cerevisiae* ITV-01 at high sugar concentrations. *Bioprocess and Biosystems Engineering*.

[42] Kasavi C., Finore I., Lama L. (2012). Evaluation of industrial *Saccharomyces cerevisiae* strains for ethanol production from biomass. *Biomass & Bioenergy*.

[43] Le Man H., Rene E. R., Behera S. K., Park H.-S. (2011). Main and interaction effects of process parameters on the ethanol production capacity of food-waste leachate by *Saccharomyces Cerevisiae*. *KSCE Journal of Civil Engineering*.

[44] Jin H., Liu R., He Y. (2012). Kinetics of batch fermentations for ethanol production with immobilized Saccharomyces cerevisiae growing on sweet sorghum stalk juice. *Procedia Environmental Science*.

[45] Ariyajaroenwong P., Laopaiboon P., Jaisil P., Laopaiboon L. (2012). Repeated-batch ethanol production from sweet sorghum juice by *Saccharomyces cerevisiae* immobilized on sweet sorghum stalks. *Energies*.

[46] Wakamatsu M., Tani T., Taguchi H., Matsuoka M., Kida K., Akamatsu T. (2013). Ethanol production from d-lactic acid by lactic acid-assimilating *Saccharomyces cerevisiae* NAM34-4C. *Journal of Bioscience and Bioengineering*.

[47] Hu N., Yuan B., Sun J., Wang S.-A., Li F.-L. (2012). Thermotolerant *Kluyveromyces marxianus* and *Saccharomyces cerevisiae* strains representing potentials for bioethanol production from Jerusalem artichoke by consolidated bioprocessing. *Applied Microbiology and Biotechnology*.

[48] Hickert L. R., Da Cunha-Pereira F., De Souza-Cruz P. B., Rosa C. A., Ayub M. A. Z. (2013). Ethanogenic fermentation of co-cultures of *Candida shehatae* HM 52.2 and *Saccharomyces cerevisiae* ICV D254 in synthetic medium and rice hull hydrolysate. *Bioresource Technology*.

[49] Pérez-Carrillo E., Cortés-Callejas M. L., Sabillón-Galeas L. E. (2011). Detrimental effect of increasing sugar concentrations on ethanol production from maize or decorticated sorghum mashes fermented with *Saccharomyces cerevisiae* or *Zymomonas mobilis*. *Biotechnology Letters*.

[50] Zhang C.-M., Jiang L., Mao Z.-G., Zhang J.-H., Tang L. (2011). Effects of propionic acid and pH on ethanol fermentation by *Saccharomyces cerevisiae* in cassava mash. *Applied Biochemistry and Biotechnology*.

[51] Harde S. M., Bankar S. B., Ojamo H., Granström T., Singhal R. S., Survase S. A. (2014). Continuous lignocellulosic ethanol production using *Coleus forskohlii* root hydrolysate. *Fuel*.

[52] Tomás A. F., Karagöz P., Karakashev D., Angelidaki I. (2013). Extreme thermophilic ethanol production from rapeseed straw: using the newly isolated *Thermoanaerobacter pentosaceus* and combining it with *Saccharomyces cerevisiae* in a two-step process. *Biotechnology and Bioengineering*.

[53] Wongwatanapaiboon J., Kangvansaichol K., Burapatana V. (2012). The potential of cellulosic ethanol production from grasses in Thailand. *Journal of Biomedicine and Biotechnology*.

[54] Taherzadeh M. J., Karimi K. (2011). Fermentation inhibitors in ethanol processes and different strategies to reduce their effects. *Biofuels*.

[55] Ludwig D., Amann M., Hirth T., Rupp S., Zibek S. (2013). Development and optimization of single and combined detoxification processes to improve the fermentability of lignocellulose hydrolyzates. *Bioresource Technology*.

[56] Jayakody L. N., Hayashi N., Kitagaki H. (2011). Identification of glycolaldehyde as the key inhibitor of bioethanol fermentation by yeast and genome-wide analysis of its toxicity. *Biotechnology Letters*.

[57] Kim S.-K., Park D.-H., Song S. H., Wee Y.-J., Jeong G.-T. (2013). Effect of fermentation inhibitors in the presence and absence of activated charcoal on the growth of *Saccharomyces cerevisiae*. *Bioprocess and Biosystems Engineering*.

[58] Parawira W., Tekere M. (2011). Biotechnological strategies to overcome inhibitors in lignocellulose hydrolysates for ethanol production: review. *Critical Reviews in Biotechnology*.

[59] Alriksson B., Cavka A., Jönsson L. J. (2011). Improving the fermentability of enzymatic hydrolysates of lignocellulose through chemical in-situ detoxification with reducing agents. *Bioresource Technology*.

[60] De Bari I., Cuna D., Di Matteo V., Liuzzi F. (2014). Bioethanol production from steam-pretreated corn stover through an isomerase mediated process. *New Biotechnology*.

[61] Zhu J., Yong Q., Xu Y., Yu S. (2011). Detoxification of corn stover prehydrolyzate by trialkylamine extraction to improve the ethanol production with *Pichia stipitis* CBS 5776. *Bioresource Technology*.

[62] Grzenia D. L., Dong R. W., Jasuja H., Kipper M. J., Qian X., Wickramasinghe S. R. (2012). Conditioning biomass hydrolysates by membrane extraction. *Journal of Membrane Science*.

[63] Wan P., Zhai D., Wang Z., Yang X., Tian S. (2012). Ethanol production from nondetoxified dilute-acid lignocellulosic hydrolysate by cocultures of *Saccharomyces cerevisiae* Y5 and *Pichia stipitis* CBS6054. *Biotechnology Research International*.

[64] Canilha L., Carvalho W., De Almeida Felipe M. D. G., De Almeida E Silva J. B., Giulietti M. (2010). Ethanol production from sugarcane bagasse hydrolysate using *Pichia stipitis*. *Applied Biochemistry and Biotechnology*.

[65] Park E. Y., Naruse K., Kato T. (2012). One-pot bioethanol production from cellulose by co-culture of *Acremonium cellulolyticus* and *Saccharomyces cerevisiae*. *Biotechnology for Biofuels*.

[66] Tesfaw A., Assefa F. (2014). Co-culture: a great promising method in single cell protein production, a review. *Biotechnology and Molecular Biology Review*.

[67] Duarte W. F., Amorim J. C., Schwan R. F. (2013). The effects of co-culturing non-*Saccharomyces* yeasts with *S. cerevisiae* on the sugar cane spirit (cachaça) fermentation process. *Antonie van Leeuwenhoek*.

[68] Vilela-Moura A., Schuller D., Mendes-Faia A., Côrte-Real M. (2010). Effects of acetic acid, ethanol, and SO_2_ on the removal of volatile acidity from acidic wines by two *Saccharomyces cerevisiae* commercial strains. *Applied Microbiology and Biotechnology*.

[69] Sankh S. N., Deshpande P. S., Arvindekar A. U. (2011). Improvement of ethanol production using *Saccharomyces cerevisiae* by enhancement of biomass and nutrient supplementation. *Applied Biochemistry and Biotechnology*.

[70] Ding J., Huang X., Zhang L., Zhao N., Yang D., Zhang K. (2009). Tolerance and stress response to ethanol in the yeast *Saccharomyces cerevisiae*. *Applied Microbiology and Biotechnology*.

[71] Chotineeranat S., Wansuksri R., Piyachomkwan K., Chatakanonda P., Weerathaworn P., Sriroth K. (2010). Effect of calcium ions on ethanol production from molasses by *Saccharomyces cerevisiae*. *Sugar Tech*.

[72] Matsushika A., Sawayama S. (2010). Effect of initial cell concentration on ethanol production by flocculent *Saccharomyces cerevisiae* with xylose-fermenting ability. *Applied Biochemistry and Biotechnology*.

[73] Laluce C., Tognolli J. O., de Oliveira K. F., Souza C. S., Morais M. R. (2009). Optimization of temperature, sugar concentration, and inoculum size to maximize ethanol production without significant decrease in yeast cell viability. *Applied Microbiology and Biotechnology*.

[74] Vilela A., Schuller D., Mendes-Faia A., Côrte-Real M. (2013). Reduction of volatile acidity of acidic wines by immobilized Saccharomyces cerevisiae cells. *Applied Microbiology and Biotechnology*.

[75] Gökgöz M., Yiğitoğlu M. (2011). Immobilization of *Saccharomyces cerevisiae* on to modified carboxymethylcellulose for production of ethanol. *Bioprocess and Biosystems Engineering*.

[76] Sembiring K. C., Mulyani H., Fitria A. I., Dahnum D., Sudiyani Y. (2014). Rice flour and white glutinous rice flour for use on immobilization of yeast cell in ethanol production. *Energy Procedia*.

[77] Kirdponpattara S., Phisalaphong M. (2013). Bacterial cellulose-alginate composite sponge as a yeast cell carrier for ethanol production. *Biochemical Engineering Journal*.

[78] Zabed H., Faruq G., Sahu J. N., Azirun M. S., Hashim R., Nasrulhaq Boyce A. (2014). Bioethanol production from fermentable sugar juice. *The Scientific World Journal*.

